# Draft genome sequence of endometrial *Olegusella massiliensis* R25 from a patient with adenomyosis

**DOI:** 10.1128/mra.01234-25

**Published:** 2026-03-11

**Authors:** Charley Holmes, Nicole R. Jimenez, Bonnie Hurwitz, Melissa M. Herbst-Kralovetz

**Affiliations:** 1 Department of Basic Medical Science, University of Arizona, College of Medicine-Phoenix 14289, Phoenix, Arizona, USA; 2 Department of Life Science, University of Bath 1555, Bath, United Kingdom; 3 Department of Obstetrics and Gynecology, College of Medicine-Phoenix, University of Arizona 42283, Phoenix, Arizona, USA; 4 Department of Biosystems Engineering, University of Arizona 8041, Tucson, Arizona, USA; 5 University of Arizona Cancer Center 613590, Tucson, Arizona, USA; Loyola University Chicago, Chicago, Illinois, USA

**Keywords:** *Olegusella*, endometrium, adenomyosis, gynecologic health

## Abstract

This report reviews *Olegusella massiliensis* strain R25 draft genome (1.86 Mb) isolated from the endometrium of a woman diagnosed with adenomyosis.

## ANNOUNCEMENT

Isolation of the KHD7 strain from the vagina of a patient with bacterial vaginosis (BV) led to reclassification within the family *Atopobiaceae*, establishing the novel *Olegusella massiliensis* (1). These gram-positive, motile coccobacilli are obligate anaerobes frequently found within the human genital tract and implicated in pathologies, such as BV (1). BV-associated *Atopobiaceae* are emerging as pathogens with adverse sequelae, including sexually transmitted infections (2), gynecologic malignancies (3, 4), and endometritis (5). This announcement examines genomic characteristics and strain relationships of *Olegusella massiliensis* R25 isolated from the human endometrium.

This study was conducted as part of a larger multi-omic study investigating endometrial cancer and benign conditions (6–9), with approval from the Institutional Review Board of the University of Arizona (reference no. 1708726047). The study participant who was diagnosed with adenomyosis provided written informed consent. Endometrial swabs were collected post-hysterectomy by swabbing bivalved uterus and frozen in Amies transport media with 10% glycerol. Isolation and bacteria culturing occurred under anaerobic conditions at 37°C on tryptic soy agar supplemented with 5% sheep’s blood for 48 h. The Qiagen DNeasy PowerSoil Pro Kit (MO BIO Laboratories, Carlsbad) was used for extraction with the cell pellet, and the resulting DNA was then sequenced at the University of Arizona PANDA Core for Genomics & Microbiome Research. Paired-end sequencing was performed using Illumina’s PCR-Free Library Prep and the NextSeq 1000 Platform (300-cycle) with read length of 35–151. Trimmomatic (v0.39) (10) improved read quality and was assessed with FastQC (v0.11.9) (11). Kraken2 (v2.1.3) and Bracken (v2.8) (12), k2_pluspf database (downloaded on 2023-06-05) (13), and Krakentools (v1.2) (12) (extract_kraken_reads.py, --taxid 9606, --include-children) were used to separate human from microbial reads. Assembly utilized Unicycler (v16.0) (14), followed by quality checks with Checkm2 (v1.0.1, -m 500) (15) and Quast (v5.2.0) (16). Annotation was performed using PGAP (v 6.1) (17). Default parameters were used for all tools, unless otherwise specified. All codes are available on GitHub (https://github.com/hurwitzlab/vaginal_genome_assembly). Genomic analyses were performed on the Bacterial and Viral Bioinformatics Resources Center website (18).

The *O. massiliensis* R25 draft genome consists of 1,861,270 base pairs, 45 contigs, a 49.18% GC content, 1,666 coding sequences (CDS), 45 tRNAs, and four rRNAs (Table 1). Among the predicted proteins, 1,658 had functional assignments, and 512 were hypothetical (Table 1).

**TABLE 1:** Genome characteristics for *Olegusella massiliensis* R25^*a*^

Parameter	Value or result for *Olegusella massiliense* R25
Isolate information	
Isolation source	Endometrium
Health status	Adenomyosis
Strain identity	
BV-BRC genome similarity	*Olsenella*/*Olegusella* sp. KHD7
Genome similarity accession number	FLLS01
K-mer count	437/1,000
K-mer distance	0.02367777
Average nucleotide identity to representative strain (%)	97.84
Genome characteristics	
Genome size (bp)	1,861,270
Number of contigs	45
Total raw reads	293,065
Sequencing depth	462×
Contig N50 (bp)	101,889
Contig L50	6
GC (%)	49.18
Genome coverage	200×
Number of 5S rRNAs	0
Number of 16S rRNAs	2
Number of 23S rRNAs	2
Number of tRNAs	45
Number of CDS	1,666
Number of CDS with functional assignments	1,658
Number of unique protein families	15
Unique protein family IDs (PGFAMs)—hypothetical	PGF_00706455, PGF_07760082, PGF_08075170, PGF_07596368, PGF_08152940, PGF_02624024, PGF_01545113, PGF_01545464, PGF_00373423, PGF_02790177, PGF_10323368, PGF_08468169
Unique protein family IDs (PGFAMs)—non-hypothetical	PGF_00060495, PGF_00413398, PGF_12832485
Number of antibiotic resistance genes (PATRIC)	18
Antibiotic resistance gene IDs	Alr, DdI, dxr, EF-G, EF-Tu, folA, folP, gidB, gyrA, gyrB, Iso-tRNA, MurA, PgsA, rho, rpoB, rpoC, S10p, S12p

^*a*^ The table includes isolate information, including the source and health status of the patient from which the isolate was obtained. Taxonomic lineage was performed with Kraken2 and verified with BV-BRC genome similarity based on k-mer distance. Average nucleotide identity is provided compared to *Olsenella*/*Olegusella* sp. KHD7 representative strain for *Olegusella massiliensis*. Annotation and genomic characteristics were identified, including genome size, number of contigs, total raw reads, contig N50, contig L50, GC content, genome coverage, number of 5S rRNA, 16S rRNA, 23S rRNA, tRNAs, CDS, and CDS with functional assignments. BV-BRC annotation and analysis also identified the number of unique protein families in the R25 genome, along with their PATRIC cross-genus family IDs, as well as the number of antibiotic resistance genes identified by PATRIC, including their corresponding gene IDs. Methods and version numbers for genome assembly can be found at https://github.com/hurwitzlab/vaginal_genome_assembly.

Subsystem classification identified metabolism (148 genes), protein processing (123 genes), energy (48 genes), and stress response, defense, and virulence (36 genes) as major functional groups (Table 1). Stress-associated pathways encompassed iron-sulfur cluster repair and NAD(P)H damage repair (Table 1). Protein comparisons to publicly available strains identified 15 families unique to R25 (Table 1). Of these, two cellular signaling genes (an ECF transporter transmembrane component and a two-component system histidine kinase) and a teichoic acid biosynthesis gene were detected. The remaining families were hypothetical (Table 1). Studies indicate microbiota influences the adenomyosis development risk (19); therefore, these genomic features may highlight its role in uterine disease.

**Fig 1:**
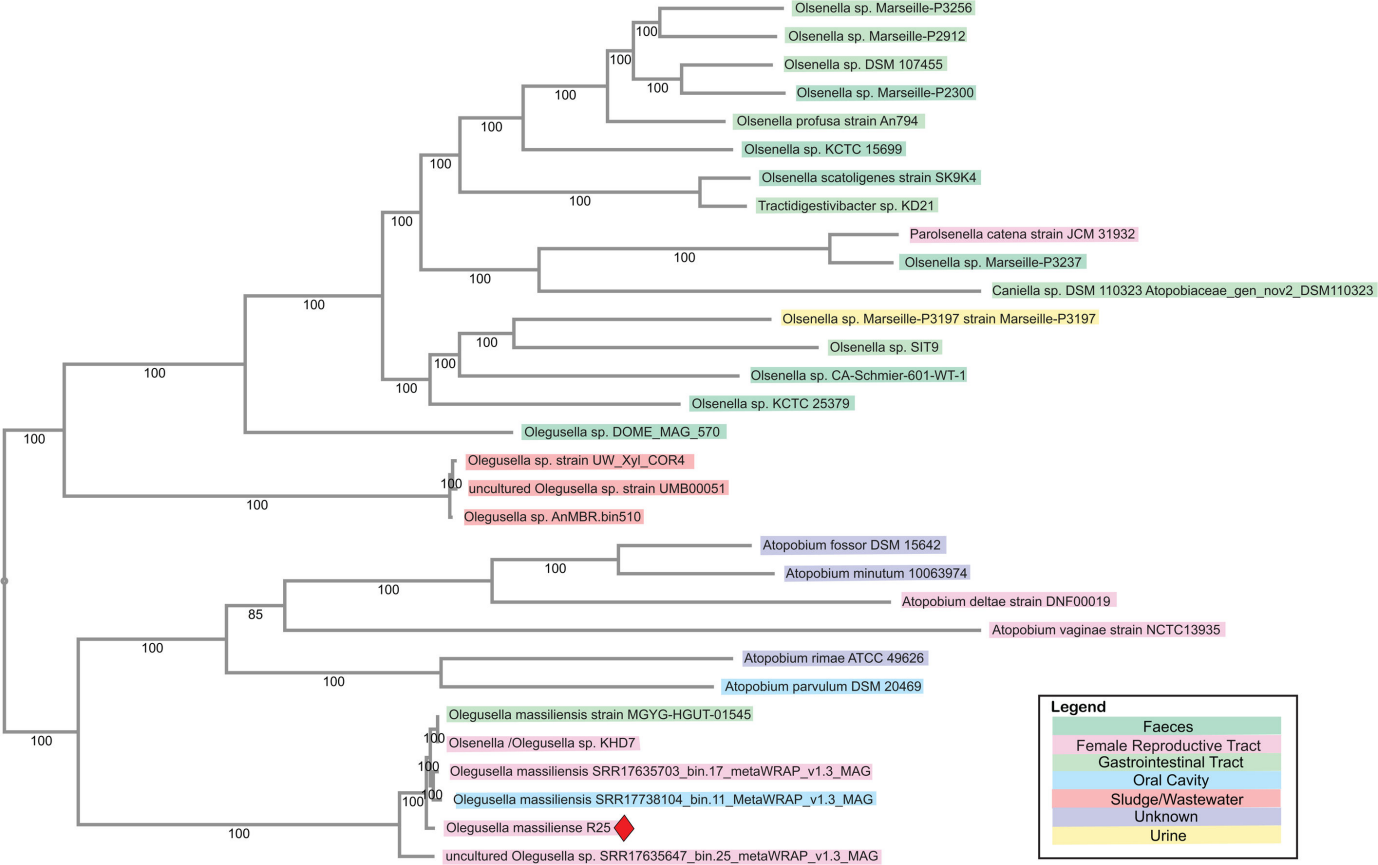
Single-copy orthologous phylogenetic tree among 30 publicly available *Atopobiaceae* strains and *O. massiliensis* R25 strain. Single-copy orthologs (*n* = 178) were used to create the tree and aligned by codon using RaXML as part of the BV-BRC bacterial genome tree pipeline, with default parameters. The tree comprises nine whole genomes and 21 metagenome-assembled genomes. In this announcement, *Olegusella massiliensis* R25 is the strain discussed in this genome announcement and indicated by a red diamond. Colors of strain names are based on the isolation source from which the genomes were collected.

Using 178 single-copy orthologs and RaxML (20) alignment, *O. massiliensis* R25 is classified within the *Olegusella* genus (Fig. 1) with an average nucleotide identity of 97.84% (Table 1). The *O. massiliensis* clade was clearly separated from the *Olsenella* and *Atopobium* species, additionally supporting *O. massiliensis*’ association with the female reproductive tract.

## Data Availability

The draft genome sequence of *O. massiliensis* R25 has been deposited at the Sequence Read Archive (SRA) (SRR35905709) under accession number NZ_JBRAVD000000000. This genome is part of larger BioProject number PRJNA1036657 associated with 16S rRNA level data of vaginal (SAMN38145775) and rectal (SAMN38145877) microbiomes, and the genome BioSample identification is SAMN51300894. The genome annotation can also be found at GenBank GCA_052772145.1; additional genome assembly, annotation, protein family, and phylogenetic analysis information at BV-BRC (https://www.bv-brc.org/workspace/jimeneznr@patricbrc.org/Olegusella). Additional assembly information and parameters utilized can be found at https://github.com/hurwitzlab/vaginal_genome_assembly.
